# Dermoscopy of circumscribed palmar hypokeratosis^⋆^

**DOI:** 10.1016/j.abd.2022.01.014

**Published:** 2023-04-20

**Authors:** Érika Hiromi Hayacibara, Sheila Zanconato Sales, Rute Facchini Lellis, Rosana Lazzarini

**Affiliations:** a Private Practice of Dermatology, São Paulo, SP, Brazil; b Dermatology Clinic, Hospital da Santa Casa de Misericórdia de São Paulo, São Paulo, SP, Brazil; c Laboratory of Pathology, Hospital da Santa Casa de Misericórdia de São Paulo, São Paulo, SP, Brazil

*Dear Editor,*

A 71-year-old female patient of Asian descent was referred to the Dermatology Outpatient Clinic for treatment of seborrheic keratoses. Two years later, in one of the consultations, an asymptomatic lesion on the left thenar region was observed and described, characterized at the time as possible excoriation. However, due to the persistence and increase in the size investigation of the lesion was initiated. The patient denied local trauma. The lesion remained asymptomatic, measuring 1.6 × 1.2 cm, characterized by a single, rounded area, with a depressed center and slightly erythematous, non-scaling, with well-defined and raised borders (Fig. 1).

**Figure 1:**
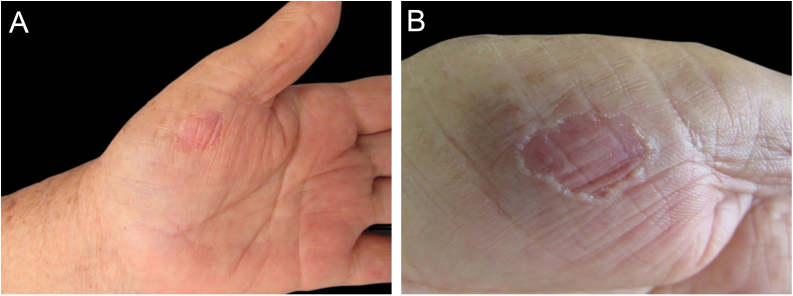
(A) Clinical record of the lesion after four years of evolution. (B) Detail of the lesion at higher magnification: dermatosis on the thenar region of the left palm, characterized by a slightly erythematous area with raised borders.

Dermoscopy was performed using the Dermlite DL4® device (3 Gen, San Juan Capistrano, USA) at level 0 (10×), without immersion gel. The central region of the lesion showed white dots, red dots and whitish striae, with regular and symmetrical distribution on a light pink background. In addition, a border with peripheral desquamation similar to a “step ladder” was observed (Figure 2, Figure 3, Figure 4). The proposed diagnostic hypotheses were Bowen's disease, porokeratosis, granuloma annulare, and self-inflicted trauma.

**Figure 2:**
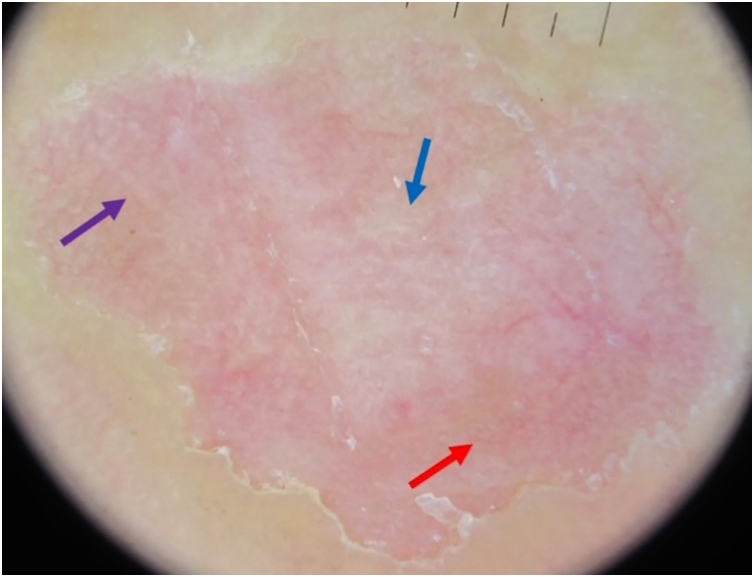
Dermoscopic image, with polarized light, showing white dots (blue arrow), red dots (red arrow) and whitish striae in the central region of the lesion.

**Figure 3:**
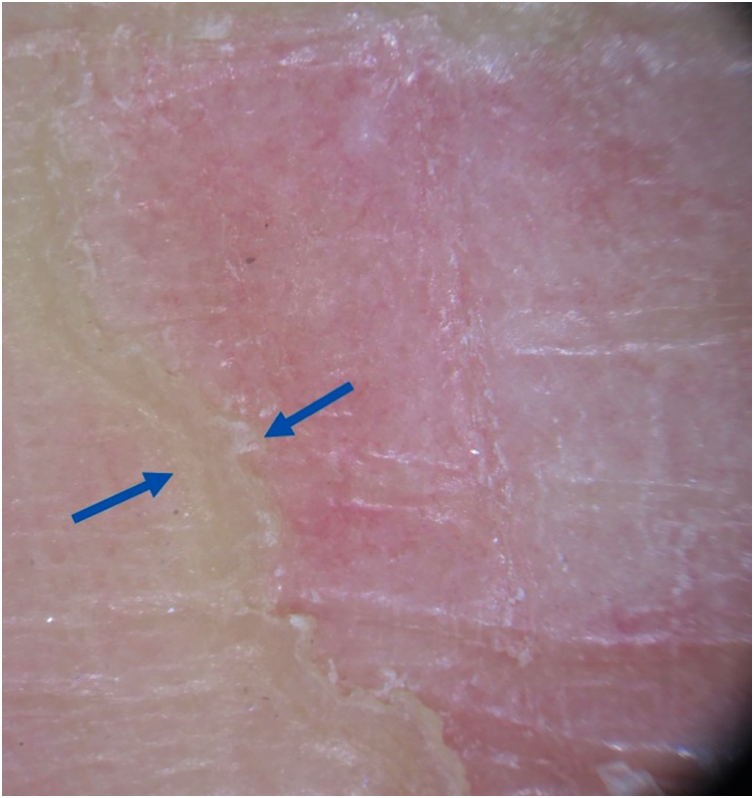
In greater detail, dermoscopic image showing a clear difference in relief between the border and the center of the lesion.

**Figure 4:**
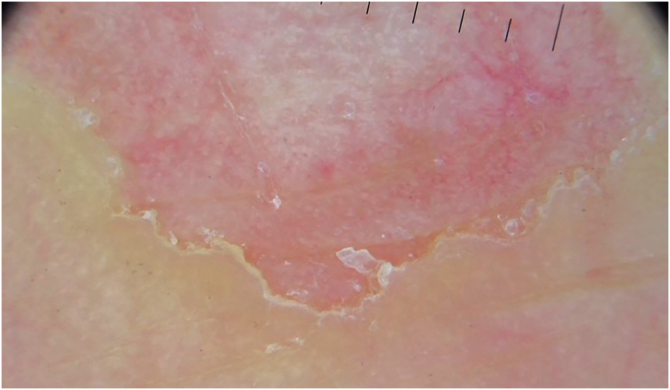
Dermoscopic image of the medial border of the lesion. At this higher magnification, it is possible to see the “step-ladder” pattern corresponding to the anatomopathological image in Fig. 5.

A skin biopsy was performed, and histopathology revealed a well-circumscribed area in which there was a decrease in the stratum corneum (Fig. 5). After evaluating the literature and considering the result of the anatomopathological examination, the authors considered circumscribed palmar hyperkeratosis. However, the patient was lost to follow-up as of the submission of this article.

**Figure 5:**
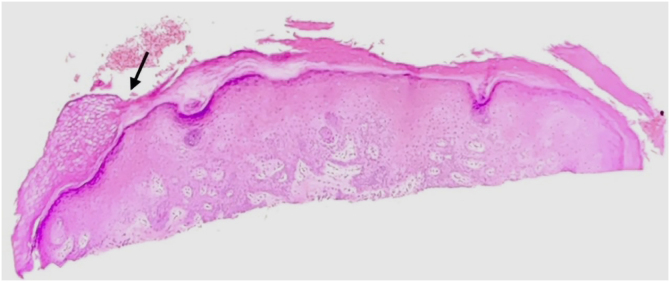
Histopathology of the lesion, showing a well-circumscribed area of clear cut decrease in the stratum corneum (arrow), (Hematoxylin & eosin, x40).

## Discussion

In 2002, Perez et al. published a series of ten cases, in which they described a new entity, characterized by solitary, asymptomatic, circular, circumscribed, erythematous and depressed palmar or plantar lesions – mainly on the thenar and hypothenar eminences of the palms. At the time, the authors described it as an epidermal malformation, calling it circumscribed palmoplantar hyperkeratosis (CPH) and reported that it is more prevalent in elderly patients and women, preferentially affecting the palms.^1^

Since then, about 100 cases have been described, but their etiology remains unknown. In 2013, Rocha & Nico described the first cases in Brazil, corroborating the main findings of Perez et al., although one of the cases had more than one lesion.^2^

The first dermoscopic description of CPH in the literature was performed by Ishiko et al.,^3^ in 2007, reporting two cases in Asian women. Since then, the reports have grown in frequency, containing descriptions similar to the case reported herein: “step-ladder-like” desquamation on the periphery of the lesion, well-demarcated erythema, with scattered white dots, which correspond to the acrosyringia, and small reddish dots, which could correspond to enlarged capillaries.^3^, ^4^, ^5^, ^6^, ^7^ Vilas Boas da Silva et al. described the presence of whitish striae in two of their three cases reported.^8^

Several etiological hypotheses have been made since the first report by Perez et al. – which suggested the possibility of a benign clonal epidermal malformation,^1^ – such as dynamic proliferative disorder, a primary disorder of keratinization located in the granular layer and stratum corneum, trauma, infection of keratinocytes by HPV, and bacterial infection such as in keratolysis plantare sulcatum.^3^

Ishiko et al. and Kanitakis et al. did not find any specific DNA or immunohistochemical markers for HPV.^3^, ^9^ However, a greater presence of Ki-67 has been described in the basal keratinocytes of the lesion than in healthy skin, as well as anti-pan keratin AE1 + AE3, except in the regions of the acrosyringium. In the suprabasal keratinocytes of the lesion, a higher expression of cytokeratin 16 was observed, with a reduction of CK-2e,^3^ CK-9 and connexin 26.^5^ This abnormal expression of keratins could explain the disease as a keratinization disorder, and even suggest the existence of subtypes.^3^, ^5^, ^9^

Urbina et al., in their literature review of 69 cases, demonstrated that most cases occur in females, over 40 years old, with a solitary lesion on the thenar region, and no previously reported trauma^7^. The patient in this report fits all this information.

Regarding the histopathological correlation, partial and abrupt loss of the stratum corneum, basket-weave orthokeratosis, capillary ectasy, and a mild inflammatory process in the superficial dermis, consistent with the erythematous background on dermoscopy, have been described. The whitish dots on dermoscopy could correspond to the acrosyringium spared from corneal loss, although Vilas Boas da Silva suggested that hyperkeratosis would explain the fact that it was more visible. There has been no description of cornoid lamella, even after the evaluation of multiple slices.^3^, ^4^, ^5^, ^6^, ^7^, ^8^, ^10^

Therefore, this report corroborates the diagnostic value of dermoscopic evaluation in CPH, since it accurately correlates with histopathology, and can help to sort out the main differential diagnoses (Bowen's disease and porokeratosis).

## Financial support

None declared.

## Authors' contributions

Érika Hiromi Hayacibara: Drafting and editing of the manuscript; collection, analysis and interpretation of data.

Sheila Zanconato Sales: Drafting and editing of the manuscript; collection, analysis and interpretation of data.

Rute Facchini Lellis: Collection, analysis and interpretation of data; effective participation in research orientation; critical review of the literature; critical review of the manuscript.

Rosana Lazzarini: Approval of the final version of the manuscript; design and planning of the study; drafting and editing of the manuscript; collection, analysis and interpretation of data; effective participation in research orientation; critical review of the literature; critical review of the manuscript.

## Conflicts of interest

None declared.
